# An Experimental Study on Botulinum Toxin Type A for the Treatment of Excessive Secretion after Submandibular Gland Transplantation in Rabbits

**DOI:** 10.1155/2016/7058537

**Published:** 2016-10-20

**Authors:** Shang Xie, Hui Xu, Bo Lin, Kan Wang, Xiao-Feng Shan, Zhi-Gang Cai

**Affiliations:** Department of Oral and Maxillofacial Surgery, Peking University School and Hospital of Stomatology, National Engineering Laboratory for Digital and Material Technology of Stomatology, Beijing Key Laboratory of Digital Stomatology, No. 22 Zhongguancun South Avenue, Haidian District, Beijing 100081, China

## Abstract

*Objectives*. To investigate whether botulinum toxin type A (BTXA) could control excessive secretion after submandibular gland (SMG) transplantation in rabbits and its possible mechanisms.* Methods*. A new SMG transplantation model was established in rabbit. 30 successfully constructed models were randomly assigned to five groups including control group and four experimental groups. Secretion outputs were used to analyze the effect of BTXA injection on excessive secretion. Hematoxylin and eosin (HE) staining, transmission electron microscopy (TEM), Western blot, and immunofluorescence were performed to analyze its possible mechanisms.* Results*. After BTXA injection, a significant decrease of excessive secretion after SMG transplantation was found in 2 and 4 weeks groups, but no significant effect on 12 and 24 weeks groups. HE and TEM results showed that BTXA led to morphological and ultrastructural changes of acinar cells of transplanted SMG. Western blot results suggested that BTXA decreased the aquaporin-5 (AQP5) protein expression after BTXA injection for 2 and 4 weeks. Immunofluorescence results showed that AQP5 protein was mainly expressed in the cytoplasm after BTXA injection for 2 and 4 weeks, which might indicate that BTXA promoted AQP5 expression from the cell membrane to cytoplasm.* Conclusion*. BTXA could effectively control excessive secretion after SMG transplantation in rabbits.

## 1. Introduction

Dry eye syndrome or keratoconjunctivitis sicca is one of the most common diseases in ophthalmology [1, 2]. According to published investigations, its prevalence ranged from 5% to 6% in the general populations, and it reached 34% in the elderly [3]. Severe cases can cause serious complications, even loss of visual acuity. In the past three decades, submandibular gland transplantation has been demonstrated as an effective method for treating severe dry eye syndrome [4–12]. However, several complications including epiphora should not be ignored. Some published papers declared that the prevalence of epiphora after SMG transplantation was about 40% [4, 13, 14]. Although several methods, including atropine gel and operation, have been used to treat epiphora, the clinical outcomes remain unsatisfied [4, 13–15]. Botulinum toxin type A (BTXA), a neurotoxic protein produced by clostridium botulinum bacteria, has been widely used in clinic to treat the excessive gland secretion including drooling, hyperlacrimation, and hyperhidrosis [16]. In 2002, Keegan and his colleagues reported that BTXA was an effective treatment for hyperlacrimation in spite of side effects [17]. After then, Yu and his colleagues also confirmed the effect of BTXA on epiphora after SMG transplantation [4, 18]. However, both of them included few samples and no further investigations were reported about its mechanisms.

In this study, we constructed a new model of SMG transplantation in rabbit and aimed to investigate the effect of BTXA on excessive secretion after SMG transplantation and its underlying mechanisms.

## 2. Materials and Methods

### 2.1. Experimental Animals

35 male New Zealand white rabbits (weighing 2.3 ± 0.2 kg) were used to establish new SMG transplantation models. 12 hours before an operation, rabbits were fasted but free to water. All experimental procedures were approved by the Institutional Ethics Committee of Animal Research (Peking University Health Science Center).

### 2.2. Reagents and Antibodies

BTXA used in this study was as gifts by Lanzhou Institute of Biological Products, China. The titer of one-unit BTXA from this institute is about equal to 3-4 units of Dysport (Ipsen, Slough, UK) and one unit of BOTOX (Allergan, Irvine, CA, USA) [19]. FITC, antibodies to aquaporin-5 (AQP5) and *β*-actin, were purchased from Santa Cruz Biotechnology (Santa Cruz, CA, USA). Other reagents and chemicals were of analytical grade.

### 2.3. Operative Technique and Experimental Groups

**Figure 1:**
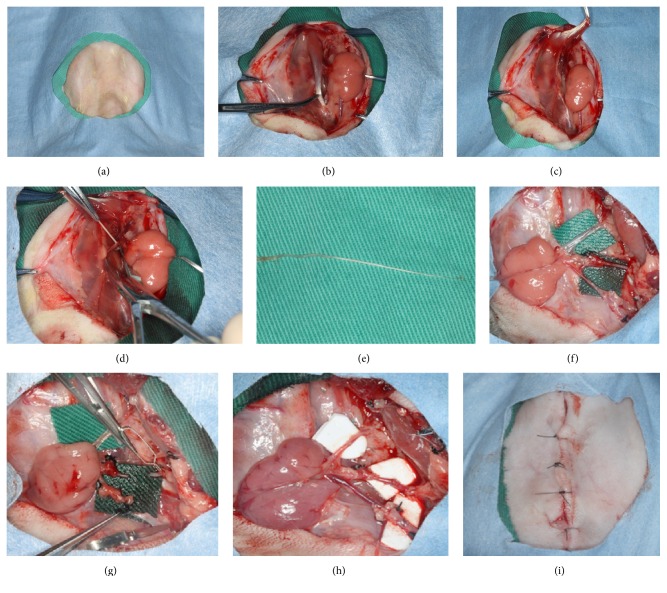
The surgical procedure of submandibular gland transplantation in animal model in rabbits ((a) skin preparation; (b) separation of anterior belly of digastric muscle; (c) cutting the digastric muscle tendon; (d) tearing the lingual nerve; (e) removing lingual nerve; (f) free submandibular gland with its blood vessels and Wharton's duct; (g) cutting the artery and vein of submandibular gland; (h) anastomosis of artery and vein in normal position; (i) suturing layer by layer).

The rabbit models of submandibular gland transplantation in previous researches were intricate and the transferred gland secretion was interrupted by rabbit accessory lacrimal gland [20–24]. To simplify and better simulate the operation, we established a novel model based on the previous models. The key operation steps were showed in Figure 1. For operation, all rabbits were anesthetized with sodium pentobarbital (20 mg/kg according to rabbits' body weight).

After three months of SMG transplantation, we detected whether the transferred SMGs were successful. During the three months, one died of disease and one was infectious. Besides, three were excluded because the SMG transplantation failed. Then, all the good transplantation models including 30 rabbits were randomly assigned to five groups (six per group), including one control group and four experimental groups (2 weeks group, 4 weeks group, 12 weeks group, and 24 weeks group). All transferred glands in the experimental groups were injected with 0.1 mL BTXA with the concentration of 100 U/mL. In experimental groups, the salivary secretion outputs were detected and the transferred SMGs were removed under anesthesia at 2, 4, 12, and 24 weeks after BTXA injection. As for the control group, salivary secretion outputs were tested and the transferred SMGs were collected after three months of SMG transplantation.

### 2.4. Measurements of Salivary Secretion

Saliva flow was measured for five minutes in rest (one minute for feeding), and we statistically calculated the length of wet filter paper with saliva (35 × 5 mm^2^) [19]. We performed the measurements of salivary secretion between 9:00 am and 12:00 am while rabbits were awake.

### 2.5. Hematoxylin and Eosin (HE) and Transmission Electron Microscopy (TEM)

To observe the morphological and structural changes, the transferred SMG sections were stained with HE and were evaluated by light microscopy. Specimens were fixed in 2% paraformaldehyde and 1.25% glutaraldehyde for assessment by TEM. Ultrathin section was stained with uranyl acetate and lead citrate and ultrastructure were acquired under a transmission electron microscope (H-7000 electron microscope; Hitachi, Tokyo, Japan).

### 2.6. Western Blot

Proteins were extracted from the transferred SMGs and quantified by BCA method as previous description [25]. 40 *μ*g proteins extracts were separated on 12% sodium dodecyl sulfate polyacrylamide gel electrophoresis and transferred to polyvinylidene difluoride (PVDF) membranes. The membranes were blocked with 5% nonfat milk for two hours and then probed with AQP5 antibody at 4°C overnight. The membranes were washed with PBST for 10 min (3 times) and then incubated with horseradish peroxidase- (HRP-) conjugated secondary antibody (1 : 4000 dilution). Finally, immunoreactive bands were visualized by enhanced chemiluminescence.

### 2.7. Immunofluorescence

Frozen sections of transferred SMG were fixed in cold acetone for 15 minutes and were immunostained with anti-AQP5 antibody (1 : 100) overnight at 4°C and incubated with FITC- or tetramethylrhodamine isothiocyanate-labeled secondary antibodies as described previously [6, 26]. Fluorescence images were captured by confocal microscope (TCS SP5; Leica, Heidelberg, Germany).

### 2.8. Statistical Analysis

Data are expressed as mean ± SE. Statistical analysis was performed by GraphPad Prism software (GraphPad Prism, La Jolla, CA, USA). Differences among groups were analyzed by one-way analysis of variance (ANOVA) and then Bonferroni testing. A value of *p* < 0.05 was considered as statistical significance.

## 3. Results

### 3.1. BTXA Could Well Control the Excessive Secretion of Transferred SMG in Rabbits

**Figure 2:**
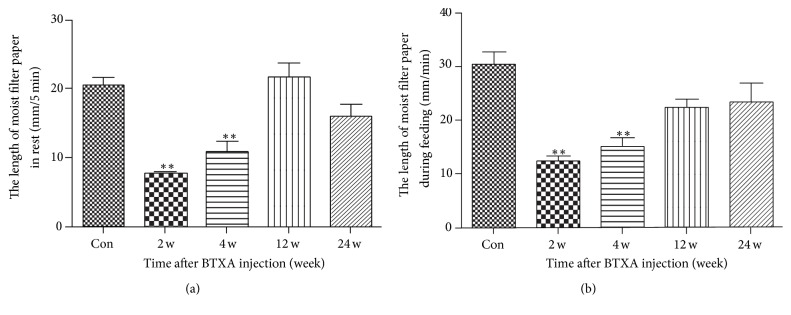
Secretion output of transplanted SMG after BTXA injection. (a) Secretion output of transplanted SMG at rest after BTXA injection in five minutes. (b) Secretion output of transplanted SMG during feeding after BTXA injection in one minute. Values are means ± SE from six independent experiments. ^*∗∗*^ *p* < 0.01* versus* control group.

To observe the role of BTXA in treating excessive secretion outputs of transferred SMG, we injected 0.1 mL BTXA with concentrations of 100 U/mL into transferred gland in the experiment groups (10 U BTXA mixed with 0.1 mL 0.9% saline). Compared with the control group, a significant decrease of secretion output was observed in the BTXA-treated group at 2 weeks and 4 weeks, whereas there was no significant difference in 12 and 24 weeks groups (Figure 2(a) for rest in 5 minutes and Figure 2(b) for feeding in one minute).

### 3.2. Histological Changes after BTXA Injection in Transferred SMG

**Figure 3:**
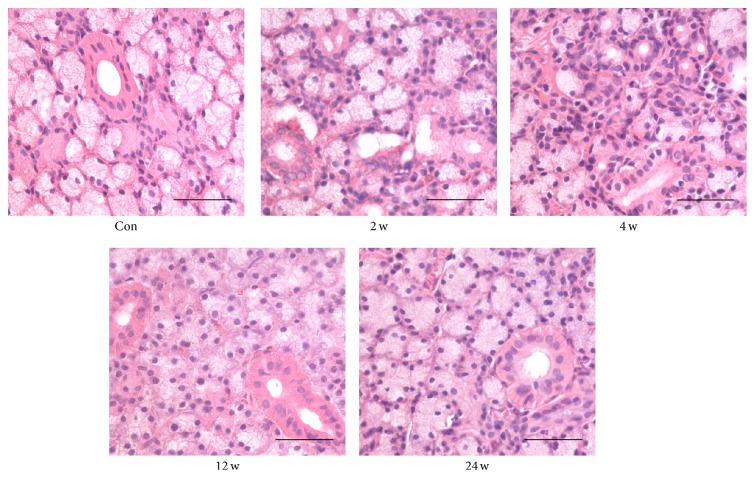
HE-stained sections for the structural changes of the transferred submandibular glands after BTXA injection on control group and 2, 4, 12, and 24 weeks groups (scale bar, 50 *μ*m).

Normal acinar and ductal cells were observed in the transferred SMG without BTXA injection (control group, Figure 3, Con). Compared with control group, the 2 and 4 weeks groups had more fibrous tissues in the glandular lobules and smaller acinar cells (Figure 3, 2 w, and Figure 3, 4 w). After 12 and 24 weeks of BTXA injection, the morphological appearance of gland recovered gradually (Figure 3, 12 w, and Figure 3, 24 w).

**Figure 4:**
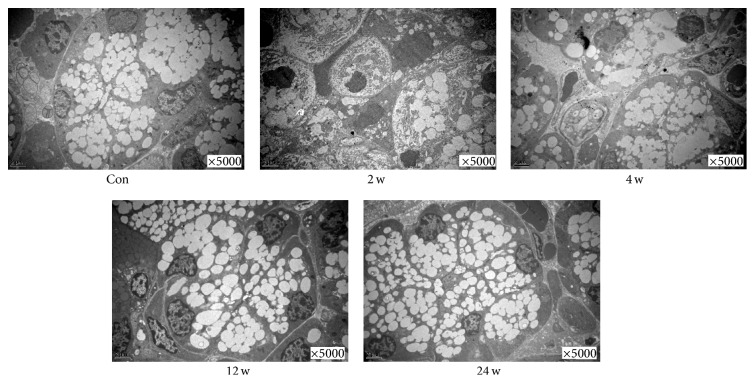
Effect of BTXA on the ultrastructure of transplanted submandibular glands. Control group and BTXA-treated groups (2, 4, 12, and 24 weeks). 5000x.

TEM indicated that massive secretory granules could be observed in the acinar cell of control group (Figure 4, Con). Compared with the control group, the volume of acinar cells was smaller and the number of secretory granules was less at the time of 2 and 4 weeks (Figure 4, 2 w, and Figure 4, 4 w). However, at 12 weeks, the morphological appearance of gland recovered gradually, and, at 24 weeks, it recovered to the normal control gland (Figure 4, 12 w, and Figure 4, 24 w).

### 3.3. BTXA Inhibited the Expression of AQP5

**Figure 5:**
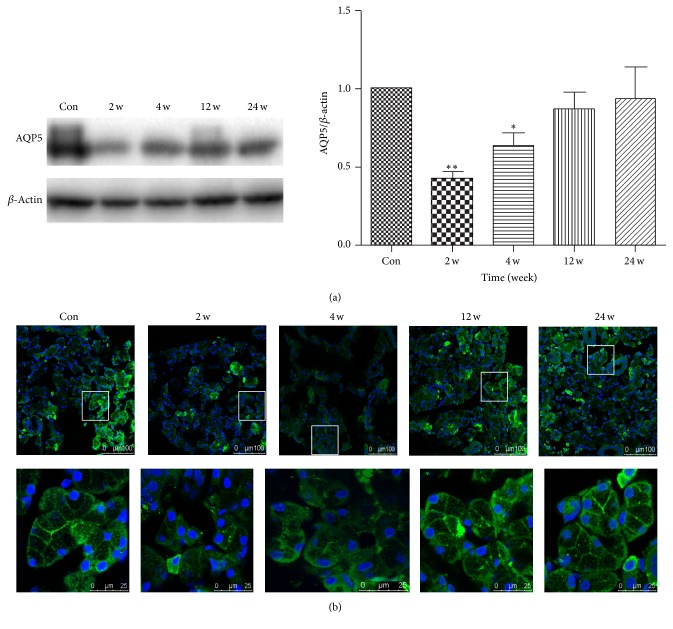
(a): Effect of BTXA on AQP5 expression in transplanted submandibular glands. Con: control group. ^*∗∗*^ *p* < 0.01 and ^*∗*^ *p* < 0.05* versus *control group; (b) effect of BTXA on AQP5 distribution in transplanted submandibular glands. Immunofluorescent localization of AQP5 (green) in control and BTXA-treated transplanted submandibular glands (2 w, 4 w, 12 w, and 24 w), with the boxed areas in the top panels (scale bar, 100 *μ*m) presented at higher magnification in the bottom panels (scale bar, 25 *μ*m). Nuclei were stained with DAPI (blue).

AQP5 is a water channel protein, which plays a role in the generation and secretion of saliva [27–29]. Proper AQP5 expression and subcellular localization are necessary to maintain water homeostasis [29, 30]. To explore whether BTXA directly affects AQP5 protein, Western blot and immunofluorescence were performed to detect the AQP5 expression and subcellular localization in acinar cells. Results suggested that AQP5 protein expression was decreased at the time of 2 weeks and 4 weeks but almost returned to the levels of control group at 12 and 24 weeks (Figure 5(a)).

### 3.4. BTXA Promoted the Translocation and Redistribution of AQP5

Immunofluorescent analysis showed that AQP5 was mainly located at the apical and lateral membranes of the acinar cells in transferred SMGs of control group (Figure 5(b), Con). In the experimental groups, results suggested that the fluorescence intensity of AQP5 reduced significantly and the AQP5 protein distributions were changed at 2 and 4 weeks group. However, all the changes almost recovered at the time of 12 and 24 weeks after BTXA injection (Figure 5(b)).

## 4. Discussion

SMG transplantation has been considered as one of the most effective treatments for severe xerophthalmia [4, 11, 31, 32]. However, about 40% of patients suffered from epiphora after SMG transplantation [13, 14, 33], which reduced patients' life quality and increased their social embarrassments. Current therapeutic strategies of epiphora after SMG transplantation include surgery, a variety of drugs, and other therapies [15, 31, 34]. However, none of them are entirely satisfactory for various side effects. Thus, it is necessary to find a better therapeutic method with safety and efficacy.

BTXA has been widely used in oral and maxillofacial surgery for almost 20 years, including treating muscular spasm, drooling, Frey's syndrome, and produced encouraging results [16, 35, 36]. Our previous animal experiments had also demonstrated that BTXA could reduce the secretory output of SMG of rabbits and rats [19, 26]. Thus, it is likely to be a good candidate for the treatment of epiphora after SMG transplantation. Although Keegan had reported that BTXA treated epiphora with good results in 2002, only one case was included and there is no high quality evidence to support its applications [17]. As is well known, drugs' safety and efficacy should be comprehensively estimated before it could be used in clinical treatment, including BTXA. Therefore, the efficacy and safety of BTXA should be well demonstrated in animal models before we could use it in patients. Considering that the previous models were intricate and did not well reflect the truth of salivary secretion [21, 22, 25, 37], we established a new transplanted SMG model in rabbits. In this model, we injected 10 U BTXA (100 U/mL) into the transferred gland and found BTXA also inhibited the salivary secretion temporarily. At the time of 2 and 4 weeks after BTXA injection, there was marked effect on glandular secretion. However, the glandular output of SMG transplantation recovered gradually at the time of 12 weeks and 24 weeks after BTXA injection, suggesting that the drug wore off over this time period, which are consistent with the previous studies [19, 26]. Besides, HE and TEM results showed atrophic and less granules of acinar cells in transferred SMGs, which might help to explain the reduced function of SMG.

It was reported that BTXA injection reduced the size of acinar cells and AQP5 expression in rabbits and rats, and AQP5 localization plays a significant role in salivary fluid secretion [19, 26, 28, 38–40]. The translocation of AQP5 from the cytoplasm to the apical plasma membrane is required to promote secretory capacity [29, 41, 42]. Besides, our previous study also found that BTXA could directly affect AQP5 expression and distribution in SMG cell without innervation, which indicates that BTXA could directly inhibit SMG cell secretion by affecting AQP5 [26]. In this study, Western blot and immunofluorescence results suggested that BTXA reduced the AQP5 protein expression and promoted AQP5 expression transposition from the cell membrane to cytoplasm after BTXA injection. Based on what was mentioned previously, we speculated that BTXA might share a similar role in the treatment of epiphora after transplanted SMG and BTXA could directly inhibit the secretion of the denervated gland.

In summary, our experiments have demonstrated that BTXA injection could effectively control the epiphora after autologous SMG transplantation in rabbits more for than 4 weeks. BTXA injection reduces AQP5 expression and promotes redistribution of AQP5 in the transplanted SMG, which suggests that BTXA inducing AQP5 protein expression reduction and redistribution might be a therapeutic target for controlling glandular secretion.
